# Enjoyment, Executive Functions, and Motor Efficiency in Youth Soccer Players: A 15-Week Observational Study

**DOI:** 10.3390/children13091204

**Published:** 2026-09-07

**Authors:** Sonia Angilletta, Enzo Iuliano, Johnny Padulo, Domenico Martone, Arianna Manavella, Goran Kuvačić, Davide Curzi, Marco Alessandria, Andrea De Giorgio

**Affiliations:** 1 Department of Theoretical and Applied Sciences, eCampus University, 22060 Novedrate, Italy; sonia.angilletta@uniecampus.it (S.A.); enzo.iuliano@uniecampus.it (E.I.); marco.alessandria@uniecampus.it (M.A.); andrea.degiorgio@uniecampus.it (A.D.G.); 2 Department of Biomedical Sciences for Health, Università degli Studi di Milano, 20133 Milan, Italy; johnny.padulo@unimi.it; 3 Department of Economics, Law, Cybersecurity and Sports Sciences, University Parthenope, 80133 Naples, Italy; 4 Department of Life Sciences and Systems Biology, University of Turin, 10124 Turin, Italy; arianna.manavella@edu.unito.it; 5 Faculty of Kinesiology, University of Split, 21000 Split, Croatia; goran.kuvacic@kifst.eu; 6 Department of Economic, Psychological, Communication, Education and Movement Sciences, University Niccolò Cusano, 00166 Rome, Italy; davide.curzi@unicusano.it

**Keywords:** enjoyment, executive functions, youth sport, soccer, children, motor performance

## Abstract

**Highlights:**

**What are the main findings?**

- During the 15-week observation period, the young soccer players showed significant changes between the beginning and end of the study in terms of motor and attentional efficiency; the other cognitive measures remained essentially unchanged.
- Perceived enjoyment was not significantly associated with cognitive or motor performance. However, enjoyment scores were consistently high, showing limited variability. This may have hindered the identification of any associations.

**What are the implications of the main finding?**

- Enjoyment and cognitive–motor performance appear to represent distinct, though potentially related, dimensions of the sports experience. They should be considered separately when evaluating training programs.
- An enjoyable training environment remains important for engagement and participation. However, enjoyment alone cannot be used as an indicator of attentional, inhibitory, or motor performance.

**Abstract:**

**Background**: Enjoyment in sport has emerged as a key psychological factor influencing exercise adherence and cognitive and emotional development. This exploratory study examined pre–post changes in selected cognitive and motor variables over a 15-week observation period and whether average enjoyment during soccer training was associated with cognitive and motor performance indices. Fifty-six players (M = 52; F = 4; age: 9.98 ± 0.70) were observed across 30 soccer training sessions over 15 weeks. **Methods**: Enjoyment was rated after each session using an emoji-based visual analogue scale, and cognitive performance was assessed before and after the observation period with tablet-based tasks. **Results**: Pre–post analyses showed significant changes in the Motor Efficiency Test (t55 = 3.15; *p* = 0.003; Δ = −19.4%; Cohen’s d = 0.42) and FlankTime (W = 1345; *p* < 0.001; Δ = −8.1%; rank-biserial correlation = 0.68). FlankAcc showed only a nominal, uncorrected distributional change (W = 323; *p* = 0.015; Δ = 0.0%). Enjoyment was not significantly associated with any of the cognitive or motor performance indices. However, enjoyment scores were clustered toward the upper end of the scale, indicating restricted variability that may have limited the ability to detect associations. **Conclusions**: These preliminary findings indicate changes in selected cognitive and motor indices over the 15-week observation period, without evidence of direct associations between perceived enjoyment and the cognitive or motor performance indices examined.

## 1. Introduction

Participation in both physical activity (PA) and sports during childhood and adolescence offers an ideal environment for motor, cognitive, and emotional development [1]. Regular PA is associated with benefits for psychological functioning and executive functions (EFs), including attentional control, inhibition, updating, and behavioral adaptation [2,3,4]. These processes are especially relevant during preadolescence, when EFs are still developing and remain sensitive to environmental, educational, and PA experiences [2,5,6]. Open-skill sports such as soccer require continuous adaptation to dynamic and unpredictable situations [7]. During the game, young athletes must select relevant information, ignore distractions, anticipate opponents’ actions, make rapid decisions, and inhibit ineffective responses [7]. Soccer therefore provides an ecologically valid context for examining EFs in youth sport [8]. This is supported by evidence linking soccer-specific performance with technical and perceptual-motor components [9], and by studies showing that more skilled young soccer players may outperform less experienced peers or non-athletes on tasks involving executive control, attention, and inhibition [10,11,12,13]. Moreover, visual skills and EFs appear to contribute jointly to soccer performance [14]. The affective and motivational quality of sport participation may also be relevant to executive functioning. Enjoyment, defined as the subjective pleasure experienced during an activity, is a key factor associated with continued participation in sport and PA [15,16]. In youth sport, enjoyment may reflect positive affect, engagement, perceived competence, social relationships, and the personal meaning attributed to the activity [17]. The theoretical rationale for linking enjoyment with EFs lies in the effect of positive emotional and motivational states on attention and cognitive control. Positive affect can broaden attentional focus and promote cognitive flexibility, although it may also increase distractibility depending on task demands [18,19]. Moreover, these effects depend on motivational intensity: low-intensity positive states tend to broaden attention, whereas approach-oriented and goal-directed positive states may narrow attentional focus and support cognitive control [20,21,22]. In youth soccer, enjoyment may therefore indicate not only well-being but also attentional and motivational engagement during training. Accordingly, dynamic soccer activities such as small-sided games have been associated with higher attention and perceived enjoyment than less playful exercise formats [23], while motivation, enjoyment, and commitment appear closely related in young soccer players [24]. Direct evidence identifying fun as an independent predictor of EFs in youth sports is limited, but the existing findings provide a relevant basis for this hypothesis. Fun, game-like sports activities can increase children’s engagement in cognitively demanding situations, thereby exposing them more frequently to contexts that require attentional skills, rapid response, effective action inhibition, and cognitive flexibility. In this sense, enjoyment should not be viewed merely as a general affective response, but also as a potential indicator of motivational engagement within activities that test executive control. Therefore, from an observational and exploratory perspective, and given the potential influence of affective and motivational states on attention and cognitive control, examining enjoyment in relation to measures of EFs could help clarify whether the subjective experience of sports is associated with individual differences in cognitive performance. Based on these considerations, and acknowledging the limited direct evidence on enjoyment as a predictor of EFs, this study had two aims: (i) to examine pre–post changes in selected cognitive variables over a 15-week soccer observation period in young soccer players; (ii) to explore whether perceived enjoyment was associated with the cognitive and motor performance indices considered in the study. Given the complex relationship between positive emotion, motivation, and cognitive control, the study adopted an exploratory approach, without assuming a causal relationship between enjoyment and cognitive performance.

## 2. Materials and Methods

### 2.1. Participants

The sample size was determined using G*Power software (version 3.1.9.6). The power analysis was conducted using a two-tailed paired-samples *t*-test with an α level of 0.05, a statistical power of 0.80, and an effect size (Cohen’s d) of 0.57 [25]. The results indicated a minimum required sample size of 27 participants. A total of 83 young soccer players were initially recruited from four soccer clubs.

**Figure 1:**
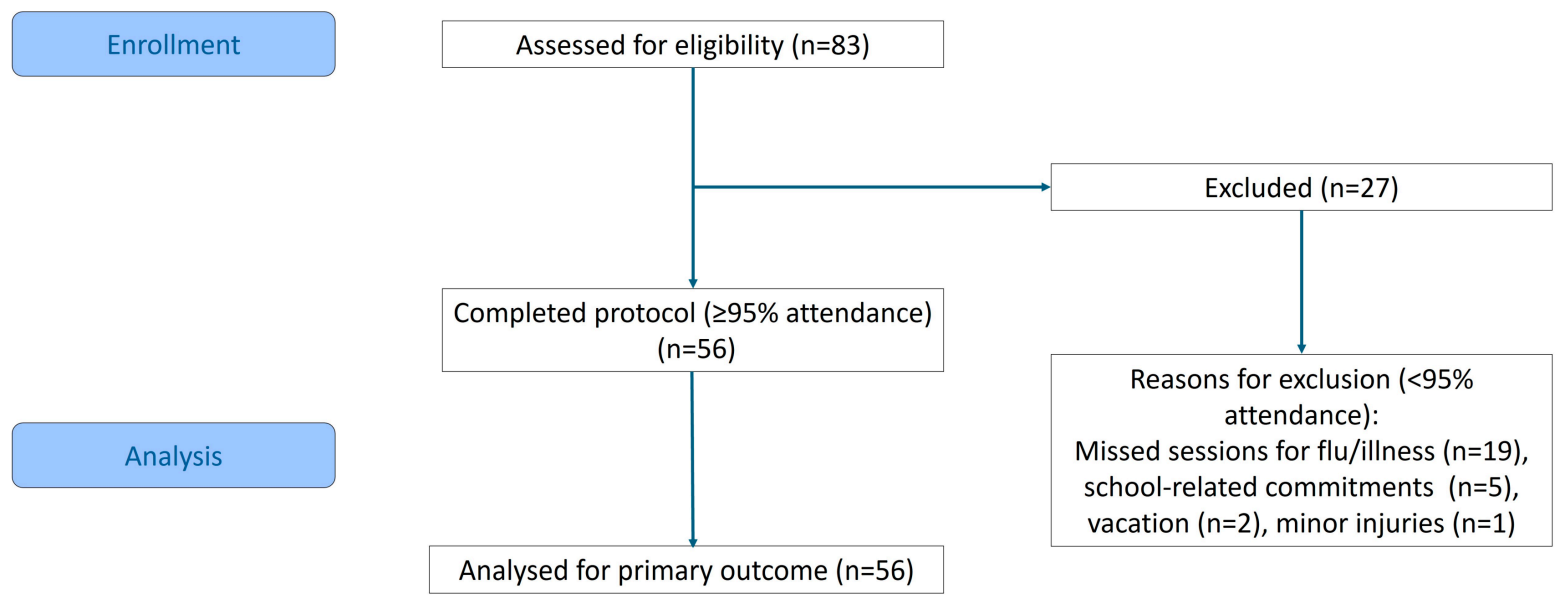
CONSORT-style flow diagram illustrating participant enrollment, exclusion criteria based on session attendance, and final sample size included in the per-protocol analysis.

Although an attendance rate of ≥95% was set as the minimum inclusion criterion, all 56 participants aged between 9 and 11 years who completed the training and data collection protocol achieved 100% attendance and were included in the final statistical analyses (F = 4; M = 52; age: 9.98 ± 0.70; body mass [kg]: 34.66 ± 8.45; height [cm]: 138.58 ± 7.76; BMI: 17.86 ± 2.93). A total of 27 participants were excluded from the analysis for failing to meet the minimum attendance threshold (<95% attendance) due to flu/illness (*n* = 19), school-related commitments (*n* = 5), vacations (*n* = 2), or minor injuries (*n* = 1). A detailed participant flow diagram illustrating enrollment, exclusions, and final sample size is presented in Figure 1.

Moreover, to be enrolled in the study, participants had to meet the following inclusion criteria: (i) be free from any diagnosis of chronic, metabolic, neurological, or other pathology that could significantly influence their lifestyle, level of enjoyment, or psychological well-being; (ii) possess a valid medical certificate for competitive physical activity and train regularly at one of the soccer clubs involved in the study; and (iii) have been playing soccer for at least two years. To avoid bias in the results, participants were excluded from the statistical analysis if they had transient or persistent conditions that could significantly influence their psychological performance or level of enjoyment, such as pharmacological treatments, injuries, or particular family circumstances. The study was designed and conducted in accordance with the principles of the Declaration of Helsinki. As all participants were underage, written informed consent was obtained from their parents, and participants and their parents were informed about the confidential handling of the coded data. The participant-coding and data-linkage procedure was included in the study protocol approved by the Unicusano University Research Ethics Committee (code: CERA02/2025; date: 13 June 2025), prior to participant enrollment and data collection.

### 2.2. Procedures

A short battery of cognitive tests was administered before (assessment 1) and after 30 training sessions (assessment 2) conducted twice a week over a period of 15 weeks. Over the 15-week period, after each of the 30 consecutive training sessions, participants were asked to rate the enjoyment they experienced during the session. All athletes completed their training sessions during the same period of the season. Everyone followed structured training that included: a warm-up phase, an athletic conditioning phase (running, guided drills), a technical phase (age–dependent, but always including ball carrying, dribbling, shooting, receiving and controlling the ball), a small-sided game, 15-min match, and penalty kicks. We also requested that coaches and parents schedule the sessions at the same time of day (i.e., from 4:00 to 5:30 pm, at an average temperature of 19 °C). This procedure was used to examine pre–post changes in cognitive and motor performance to test whether average enjoyment during training was associated with the cognitive and motor performance indices considered in the study, without attributing the observed changes to training exposure or intensity. All cognitive tests and the methods used to assess participants’ enjoyment are described in the following section. Session rating of perceived exertion has been proposed as a simple and ecologically valid method for monitoring training load in young athletes [26].

### 2.3. Cognitive Tests

The cognitive tests were administered electronically using a tablet with the CogniFit application v.4.7.8 (CogniFit Inc.; San Francisco, CA, USA) installed. Three tests were used: Eriksen’s Flankers Test, Stop-Signal Test, and Visual Search Test. Each test generated two scores: (i) mean response time for correct trials (ms; lower values indicate better performance), and (ii) accuracy (% correct; higher values indicate better performance). All cognitive assessments were conducted according to standardized procedures. The tests were administered at the end of the training session, and after enjoyment assessments, in a quiet, enclosed room separate from the locker rooms, using the same tablet model and the same version of the CogniFit app for all players. Participants received the same standardized instructions before each task and completed the practice phase provided by the app before beginning the test phase. The order of the cognitive tests was fixed as listed above and remained consistent across participants, as well as between the pre-test and post-test assessments. The assessments were administered by qualified sports psychologists who followed the same testing protocol. During the tests, the children were assessed individually, and efforts were made to minimize external distractions and interactions with peers or coaches.

### 2.4. Eriksen’s Flankers Test

Eriksen’s “flankers” test [27] involves presenting a series of five elements. The one in the center represents the target stimulus, while the four elements on either side, two on each side, constitute the “flankers.” The flankers may be congruent with the target (when they point in the same direction), incongruent (when they point in the opposite direction), or neutral (when they are not associated with either of the two possible responses). In each trial, the participant must determine whether the central stimulus is oriented to the right or to the left. After an initial familiarization phase, the test consists of 36 trials, evenly distributed across the three conditions: 12 congruent, 12 incongruent, and 12 neutral. The orientation of the targets and flankers is counterbalanced according to the criteria adopted during the learning phase, while the order of stimulus presentation is randomized. The task allows for the assessment of various cognitive components, including selective attention, executive control, inhibitory control, reaction times, processing speed, and spatial perception. Performance is summarized using two indices: (i) FlankTime, which measures efficiency in seconds; and (ii) FlankAcc, which corresponds to the percentage of correct responses.

### 2.5. Stop-Signal Test

In the Stop-Signal test [28], participants perform a series of trials designed to establish an automatic motor response. Two circles are displayed on the screen, and in each trial, one of them changes color to yellow. The task is to select the yellow circle as quickly as possible. Once this response pattern has been established, a stop signal is introduced: in some trials, the circle that was initially yellow turns red, signaling to the participant that they must stop the response and therefore avoid selecting it. After a preliminary learning phase, two gray circles are presented on the screen, one on the right and one on the left. In each trial, one of the two turns yellow, while the other remains gray. In some trials, however, the yellow circle subsequently changes color to red, signaling the need to inhibit the previously established response. The stop signal appears in 12 of the 36 trials, with three different time intervals: four times after 100 ms, four after 200 ms, and four after 300 ms. The distribution of these intervals across the No-Go trials is random. The test is primarily designed to assess response inhibition ability. The two scores provided by this test were labelled StopTime (efficiency in seconds) and StopAcc (percentage accuracy).

### 2.6. Visual Search Test

The Visual Search Test [29,30] involves identifying a target letter within a grid containing numerous alphabetic stimuli. The letter to be found is displayed on the left side of the screen and appears twice in the grid shown on the right. For each trial, the participant has 10 s to identify and select one of the two targets as quickly as possible, distinguishing it from distractor letters that vary in their degree of similarity. After an initial learning phase, the target letter is presented inside a circle on the left side of the screen, while a 10 × 14 grid appears on the right, consisting of a total of 140 black Latin letters. The composition of the grid varies depending on the target: when the target letter is “C,” the distractors are G, O, and Q, while M, W, and Z serve as filler letters; when the target is “N,” the distractors are M, W, and Z, and the filler letters are G, O, and Q. The task is primarily used to assess visual scanning ability. Performance is expressed through two indices: VisualTime, which measures time efficiency in seconds, and VisualAcc, which corresponds to the accuracy percentage.

### 2.7. Enjoyment Assessments

Enjoyment was assessed using an emoji-based visual Likert scale, adapted from child-friendly smiley-face rating methods designed to facilitate quantitative self-assessment in children aged 9 to 11 [31]. These emoji-based scales have also demonstrated psychometric acceptability for assessing affective responses in children [32]. The scale included six emojis representing increasing levels of happiness, ranging from 1 (“not happy at all”) to 6 (“very happy”). At the end of each of the 30 training sessions, each child selected the emoji that best reflected how they had felt during the session and placed the corresponding slip of paper into a collection box. Each slip of paper had a unique participant code printed on the back, allowing the research team to link repeated assessments from the same participant across different sessions, while maintaining the confidentiality of the responses from peers and coaches. The final score was calculated as the average of the ratings collected across all 30 training sessions. Since only participants who completed the entire training and data collection protocol were included in the final analyses, each participant’s score was based on the same number of ratings (i.e., all participants included in the final analyses provided a rating for each of the 30 sessions). The cited studies support the use of smiley-face and emoji-based assessment approaches as appropriate measures for children to evaluate affective responses [31,32]. However, this specific application, repeated session by session, has not been independently validated as a sport-specific measure of enjoyment. Consequently, the scale was used in the present study as a brief ecological indicator of perceived enjoyment during the session rather than as a validated measure of soccer-specific enjoyment.

### 2.8. The Motor Efficiency Test (TEM)

The Motor Efficiency Test (TEM) was developed by the Italian National Olympic Committee as a field-based, game-like assessment of motor abilities in young athletes [33]. It consists of a structured circuit with four stations and four different locomotor tasks, each requiring specific motor skills. Performance is evaluated based on both the speed of task completion and the accuracy of movement execution. The test does not exclusively reward faster performance, as children who complete fewer stations with greater precision may receive scores comparable to those of faster participants. In this way, the TEM provides an integrated measure of both quantitative and qualitative aspects of motor performance. Because the task is performed against time and requires continuous movement across different stations, it can be considered a dynamic and ecologically valid assessment of motor efficiency in children. Its structure makes it suitable for youth sports contexts, where coordination, movement control, speed, and accuracy are important components of performance.

### 2.9. Statistical Analysis

Paired-samples *t*-tests or Wilcoxon signed-rank tests were used to compare pre-test and post-test values, depending on the distribution of each variable, which was assessed using the Shapiro–Wilk normality test. Cohen’s d was calculated for paired-samples *t*-tests. Moreover, the rank-biserial correlation was calculated for the Wilcoxon signed-rank test. It should be noted that Cohen’s d (based on standard deviation units, unbounded) and the rank-biserial correlation (ranging from −1 to 1) operate on different mathematical metrics and are not directly comparable in magnitude. Effect sizes were interpreted according to Cohen’s conventional guidelines [34]. Percentage change (Δ) was calculated only for variables showing significant pre–post differences, using the following formula: (final value − initial value)/(initial value)*100.

In order to control for Type I error across the seven pre–post comparisons, a Bonferroni correction was applied, establishing an adjusted significance threshold of α = 0.007. Spearman’s rank correlation coefficients were used to examine the exploratory associations between average enjoyment across the 30 training sessions and all cognitive and motor performance indices considered in the study. For each cognitive and motor variable, the score entered in the correlation analysis was calculated as the average of the pre-test and post-test values. These analyses were exploratory and were intended to examine associations between overall perceived enjoyment and overall performance levels, rather than to model individual change from pre-test to post-test. To contextualize these correlational analyses, a post-hoc sensitivity analysis revealed that with a sample size of n = 56, α = 0.05, a statistical power of 0.80, and two-tailed, the study was powered to detect minimum effect sizes of |*r*| ≥ 0.365. Consequently, observed correlation coefficients below this threshold fall within a range that the study was not formally powered to detect.

Given the exploratory nature of these analyses, the results were interpreted cautiously, with attention to both the magnitude and statistical significance of the correlation coefficients. The presence of outlier values was checked using the interquartile range method to avoid unusual influences on the correlation analyses. No outliers were removed, as their exclusion did not change the correlation results. Furthermore, to check the robustness of the findings against potential sex-related confounds, a sensitivity analysis was performed by re-running the primary statistical tests on the male subsample alone. Data were analyzed using SPSS v.29.0.1.0 (IBM Corp., Armonk, NY, USA).

## 3. Results

The descriptive statistics (expressed as mean ± standard deviation or median [interquartile range]) of the variables considered for the pre–post comparison are summarized in Table 1.

**Figure 2:**
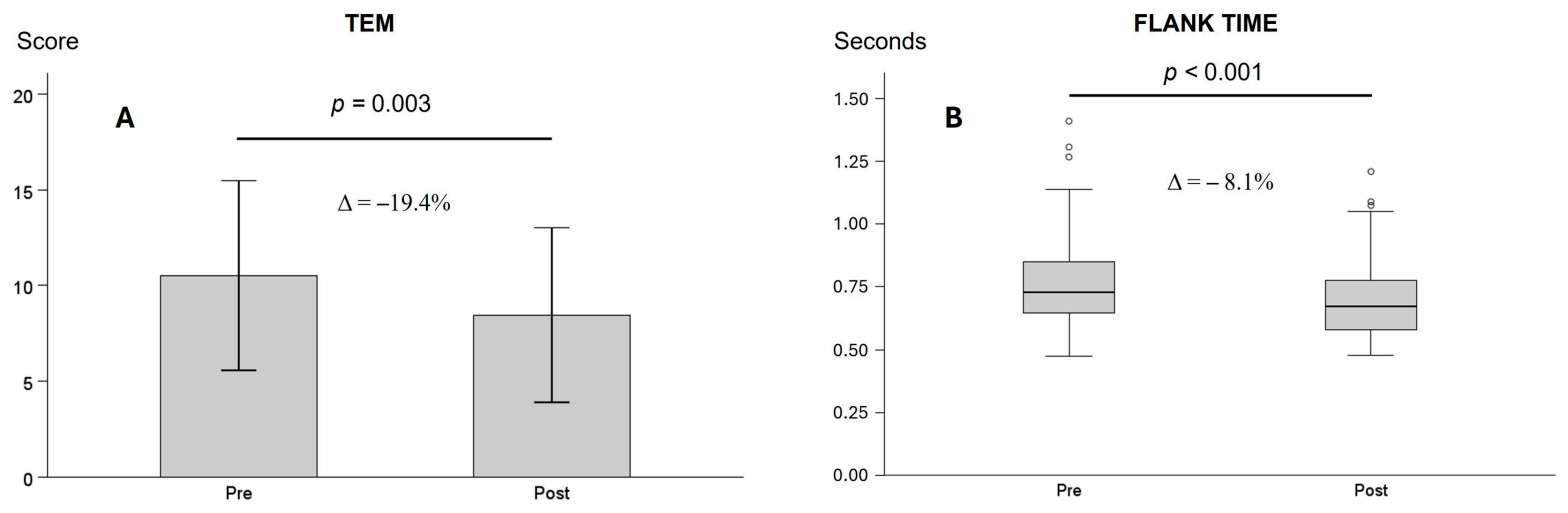
Significant differences between before (Pre) and after (Post) in the TEM and Flank variables. Panel (**A**) displays mean and standard deviation (SD) error bars, corresponding to the parametric paired-samples *t*-test used for normally distributed data. Panel (**B**) displays a box plot showing median, interquartile range (IQR), and outliers (open circles), corresponding to the non-parametric Wilcoxon signed-rank test used for non-normally distributed data. Δ indicates percentage change.

**Figure 3:**
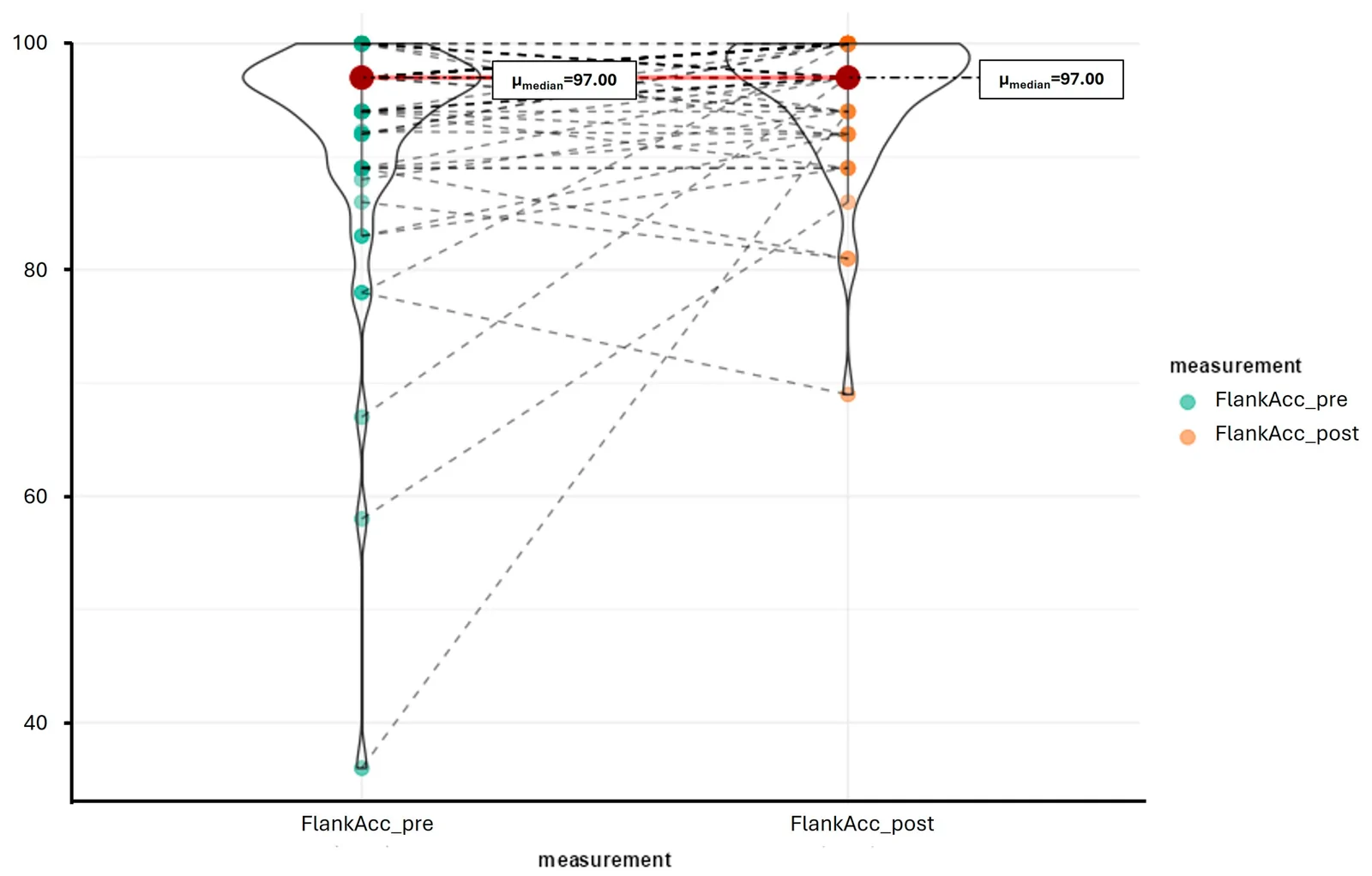
Paired violin dot-plot illustrating individual participant changes in Flankers Accuracy (FlankAcc) from Pre- to Post-assessment. Colored dots represent individual data points, dashed lines indicate paired individual trajectories, and large dark circles with connecting red lines display the median scores.

The paired-samples *t*-test showed a significant pre–post difference in TEM score (t55 = 3.15; *p* = 0.003; Δ = −19.4%; Cohen’s d = 0.42 [CI95: 0.15, 0.69]). The Wilcoxon signed-rank test showed a significant pre–post difference in FlankTime (W = 1345, *p* < 0.001; Δ = −8.1%; rank-biserial correlation = 0.68 [CI95: 0.51, 0.81]; Figure 2). StopAcc (W = 386, *p* = 0.038; Δ = 3.3%; rank-biserial correlation = −0.34 [CI95: −0.57, −0.05]) and FlankAcc (W = 323, *p* = 0.015; Δ = 0.0%, with 10 tied pairs, suggesting a difference in the tail distribution—Figure 3) showed nominal pre–post differences. However, after applying the Bonferroni correction for the seven pre–post comparisons, only TEM and FlankTime remained statistically significant at the adjusted threshold of α = 0.007.

Spearman’s rank correlation analyses did not show significant associations between enjoyment and any of the cognitive or motor performance indices considered. Table 2 summarizes the correlation coefficients and *p*-values.

Each participant’s enjoyment score was calculated as the average of the 30 ratings collected across the training sessions. Descriptive statistics indicated high overall perceived enjoyment, with a median score of 5.26 (IQR = 0.79), suggesting that enjoyment scores were clustered toward the upper end of the scale. Table 3 shows the results obtained for the non-significant variables.

The sensitivity analysis excluding the four female participants yielded a pattern of findings consistent with the main analysis, with all remaining cognitive metrics and enjoyment correlations maintaining their previous results (detailed statistical outputs are provided in the Supplementary Materials). Specifically, the pre–post improvement in FlankTime retained statistical significance after Bonferroni correction, whereas the change in TEM did not reach the adjusted significance threshold (*p* = 0.012; adjusted α = 0.007).

## 4. Discussion

Our study examined pre–post changes in selected cognitive variables over a 15-week observation period in young soccer players and assessed whether the enjoyment they experienced during training sessions was associated with cognitive performance. The results showed significant pre–post differences for TEM and FlankTime, indicating changes in these variables over the observation period. By contrast, the finding for FlankAcc should be interpreted cautiously, as it was only nominally significant and did not survive correction for multiple comparisons. These findings suggest that, in the sample analysed, certain cognitive indices changed over the 15-week observation period, but these changes do not appear to be directly associated with the level of enjoyment perceived during training sessions. This issue is particularly relevant because enjoyment scores showed evidence of a restricted range/ceiling effect, with scores clustered toward the upper end of the scale. Consequently, this limited variability may have reduced the ability to detect significant associations between enjoyment and the cognitive and motor performance indices examined.

### 4.1. Cognitive and Motor Performance Changes

It is important to emphasize that these differences between the pre-test and post-test phases should not be interpreted as evidence that soccer training led to cognitive improvement. Since the study did not include a control group or random assignment, the observed changes could also reflect developmental maturation, seasonal effects, regression to the mean, repeated exposure to the assessment protocol, familiarity with the cognitive tasks performed on tablets, greater familiarity with the assessment environment, nonspecific effects of regular sports practice, or other uncontrolled factors. From an exploratory perspective, these pre–post differences may be considered in relation to the cognitive demands of soccer, an open-skill sport in which young athletes must continuously adapt to dynamic stimuli, select relevant information, inhibit non-functional responses, and make rapid decisions under conditions of uncertainty. These demands involve executive processes such as selective attention, processing speed, and inhibitory control [35]. It is important to stress that the change observed in FlankTime concerns a task involving stimulus selection and interference control. Therefore, the present design does not allow this change to be attributed to soccer-specific cognitive demands. In particular, developmental maturation and test–retest or practice effects cannot be separated from changes potentially related to regular soccer participation. Moreover, the use of only two assessment points further prevents examination of the temporal trajectory of these changes or of any potential dose–response pattern. Previous studies have shown that young soccer players with higher levels of experience or skill may perform better on tasks involving EFs, attention, and inhibitory control [10,11,12,13]. However, these interpretations remain preliminary because the design of the present study does not allow causal attribution of the observed changes to soccer training. The result for FlankAcc requires particular caution and should not be interpreted as evidence of a general improvement in accuracy. Although the pre–post difference was nominally significant, it did not survive Bonferroni correction for multiple comparisons. Moreover, the median change was zero, suggesting that the statistically detected difference does not reflect a clear central shift in performance. Rather, the result likely reflects changes in the distribution of scores, particularly in the tails, or heterogeneous responses among participants. This pattern may also be consistent with restricted variability or ceiling effects, which are common in accuracy-based measures when performance is already high. Therefore, FlankAcc should be described as showing a nominal, uncorrected distributional change rather than a reliable improvement in attentional accuracy. In contrast, FlankTime appears to provide a more interpretable and sensitive index of change in attentional efficiency in the present sample. With regard to the sensitivity analysis conducted on the male subsample, the overall picture was fairly consistent with the analysis of the entire sample. FlankTime remained statistically significant after Bonferroni correction (*p* < 0.001; corrected α = 0.007), whereas TEM retained a similar pattern of change but no longer met the adjusted significance threshold (*p* = 0.012). FlankAcc (*p* = 0.034) and StopAcc (*p* = 0.026) showed nominal, uncorrected differences between the pre- and post-phases, while StopTime (*p* = 0.206), VisualTime (*p* = 0.431), and VisualAcc (*p* = 0.843) showed no significant differences. Therefore, among the comparisons, only FlankTime passed the Bonferroni correction in the male subsample. Given the very small number of female participants and the resulting reduction in sample size, these results should be interpreted with caution and do not allow for significant conclusions regarding sex-related differences.

### 4.2. Enjoyment and Cognitive and Motor Performance

In line with our exploratory objective, the correlation analyses revealed no statistically significant associations between enjoyment and the cognitive or motor performance indices examined (rho = 0.037 to 0.197). However, this finding should be interpreted strictly within the limits of the study’s statistical power. With a sample size of n = 56, the study was powered to detect only moderate-to-large associations (|*r*| ≥ 0.365). Consequently, these results indicate a lack of a statistically detectable moderate-to-large association at the available power, rather than a structurally established absence of any relationship.

Enjoyment is considered an important variable in youth sports, particularly as it supports motivation, participation, and continuity in PA [15,16]. Among young soccer players, enjoyment of the activity has also been linked to athletic commitment and the quality of the motivational experience [24]. However, the present findings suggest that the enjoyment perceived during training is not necessarily associated with performance on the cognitive tasks examined. The absence of significant associations should also be interpreted in light of potential individual differences in terms of exercise intensity, fatigue, and training requirements. This does not diminish the value of enjoyment in youth sports. Rather, it suggests that enjoyment may be more closely related to the subjective quality of the sports experience, persistence in practice, and overall engagement, while its relationship with specific measures of executive performance may depend on unmeasured factors such as exercise intensity, cumulative load, and fatigue. This interpretation is consistent with the broad and subjective nature of enjoyment, which can include satisfaction, engagement, a sense of competence, relationships with teammates, the relationship with the coach, satisfaction with one’s own performance, and the meaning attributed to the session. This complexity makes enjoyment an ecologically rich construct, but one that is also difficult to link linearly to individual cognitive measures. The lack of significant associations may also be partly related to the measurement approach. Averaging a brief emoji-based assessment across sessions may have reduced intra-subject variability and may not have captured distinct components of enjoyment, such as affective enjoyment, perceived competence, social interaction, or task engagement.

For example, children might report the same level of enjoyment for different reasons. One might feel this way because they believe they played well, another because they had fun with the group, and yet another because they found the training less tiring or more rewarding. These components might have different relationships with attention, reaction times, and inhibitory control. This is consistent with the literature showing that the effects of positive affect on cognitive control are not straightforward. Positive affect can broaden the scope of attention and promote cognitive flexibility, but it can also increase distractibility when the task requires careful stimulus selection [18,19]. Other studies have suggested that the effect of positive states depends on motivational intensity: approach-oriented positive states may promote a narrower focus, while those less goal-oriented may broaden the scope of attention [20,21,22]. In this study, however, enjoyment was assessed at the end of training sessions rather than during the cognitive tests. Therefore, it is plausible that it reflects the overall quality of the sporting experience rather than the attentional state active during cognitive assessment.

### 4.3. Practical Implications

From a practical perspective, these results require a balanced interpretation. Youth soccer can provide an environment rich in cognitive stimuli, especially when sessions include dynamic situations, decision-making tasks, themed games, and activities requiring selective attention and action control. However, the enjoyment derived from the activity should not be considered a direct indicator of changes in cognitive performance, and although it remains essential for sustaining motivation, adherence, and the quality of the sporting experience, it cannot be used on its own as an indicator of attentional or inhibitory performance. From this perspective, coaches and professionals should view enjoyment and cognitive performance as distinct, although potentially interrelated, dimensions of the youth sports experience. Maintaining an enjoyable training environment remains important in its own right, particularly to foster engagement and continued participation, rather than because greater enjoyment necessarily corresponds to better performance. Consequently, the evaluation of youth training programs could benefit from considering subjective experience alongside measures of cognitive and motor performance, rather than as a substitute for them.

## 5. Limitations of the Study

This study has several limitations. First, the absence of a control or comparison group substantially limits its internal validity and precludes a causal interpretation of the pre–post changes. These changes could reflect soccer training, but they could also reflect age-related cognitive and motor maturation, which is particularly relevant in the 9–11-year age range, seasonal effects, regression to the mean, repeated exposure to the testing protocol, familiarity with tablet-based activities, increased familiarity with the testing environment, nonspecific effects of regular sports participation, or other uncontrolled factors. Second, the sample was predominantly male, with only four female players included, which limits generalizability to young female soccer players and precludes a meaningful examination of sex-related differences. Third, training intensity and accumulated training load were not directly monitored. Although the sessions followed a comparable structure, internal and external demands may have varied among players and across sessions, influencing both enjoyment and cognitive performance through fatigue, perceived exertion, technical engagement, or attentional demands. Future studies should include the session RPE scale, heart rate monitoring, GPS tracking, accelerometry, or other indicators of exercise load. Fourth, enjoyment scores were clustered toward the upper end of the scale, resulting in restricted interindividual variability. This may have reduced the ability to detect (oppure statistical power to detect) associations with cognitive and motor outcomes. This limitation is particularly relevant to the interpretation of the correlational analyses. Finally, potentially relevant psychological and contextual variables (including anxiety, stress, sleep quality, technical skill level, perceived competence, motivational climate, biological maturity, and rater blindness) were not fully accounted for. Future studies should include comparison groups, workload monitoring, more detailed psychological measures, and longitudinal or experimental designs to clarify the relationship between soccer practice, enjoyment, and EFs.

## 6. Conclusions

In conclusion, this exploratory study showed preliminary pre–post changes in selected cognitive and motor variables over a 15-week observation period, but did not reveal significant associations between enjoyment and cognitive performance, although the restricted variability in enjoyment scores limits the interpretation of these correlational findings. Given the absence of a control or comparison group, these changes should not be interpreted as evidence that soccer training produced improvements in EFs. Their relationship appears complex and requires interpretive models capable of integrating cognitive, motivational, emotional, and contextual factors.

## Figures and Tables

**Table 1:** Descriptive statistics for the variables evaluated in the pre–post comparison. Data are expressed as mean and standard deviation for normally distributed variables, and as median and interquartile range (IQR) for non-normally distributed variables.

	TEM		FlankTime		FlankAcc		StopTime		StopAcc		VisualTime		VisualAcc	
	Pre	Post	Pre	Post	Pre	Post	Pre	Post	Pre	Post	Pre	Post	Pre	Post
**Mean**	10.52	8.46	/	/	/	/	/	/	/	/	/	/	/	/
**Median**	/	/	0.73	0.67	97.00	97.00	0.50	0.49	87	90	7.4	6.83	100	100
**Standard deviation**	4.95	4.56	/	/	/	/	/	/	/	/	/	/	/	/
**Interquartile range (IQR)**	/	/	0.19	0.19	5.75	6	0.09	0.14	8	10	3.98	4.33	25	25

**Table 2:** Spearman’s rank correlation coefficients between enjoyment and cognitive and motor performance indices.

	TEM		FlankTime		FlankAcc		StopTime		StopAcc		VisualTime		VisualAcc	
**Enjoyment**	Rho	*p*-value	Rho	*p*-value	Rho	*p*-value	Rho	*p*-value	Rho	*p*-value	Rho	*p*-value	Rho	*p*-value
	0.093	0.496	0.159	0.243	0.177	0.191	−0.037	0.786	0.197	0.146	−0.082	0.550	−0.087	0.523

**Table 3:** Wilcoxon W statistics and *p*-values for variables that did not show nominal pre–post differences before correction for multiple comparisons.

Variable	Wilcoxon’s W	*p*-Value
VisualTime	948	0.223
VisualAcc	165	0.966
StopTime	946	0.230

## Data Availability

The original data on which the study’s results and conclusions are based may be provided by the authors upon request.

## Supplementary Materials

The following supporting information can be downloaded at https://www.mdpi.com/article/10.3390/children13091204/s1. S1. Sensitivity analysis (n = 52 males): Pre-to-post intervention changes in cognitive metrics. S2. Spearman rank-biserial correlation analysis between physical enjoyment and cognitive performance metrics.

## Abbreviations

The following abbreviations are used in this manuscript:

TEM	Motor Efficiency Test
RPE	Rate of Perceived Exertion
GPS	Global Positioning System
IQR	Interquartile Range
